# Cardiac regeneration: Options for repairing the injured heart

**DOI:** 10.3389/fcvm.2022.981982

**Published:** 2023-01-12

**Authors:** Jun Wang, Meilin An, Bernhard Johannes Haubner, Josef M. Penninger

**Affiliations:** ^1^Department of Medical Genetics, Life Sciences Institute, The University of British Columbia, Vancouver, BC, Canada; ^2^Department of Internal Medicine III (Cardiology and Angiology), Innsbruck Medical University, Innsbruck, Austria; ^3^Department of Cardiology, University Heart Center, University Hospital Zurich, Zurich, Switzerland; ^4^Institute of Molecular Biotechnology of the Austrian Academy of Sciences, VBC – Vienna BioCenter, Vienna, Austria

**Keywords:** cardiac regeneration, cell-free therapies, cell-based therapies, hPSC-CMs, transplantation

## Abstract

Cardiac regeneration is one of the grand challenges in repairing injured human hearts. Numerous studies of signaling pathways and metabolism on cardiac development and disease pave the way for endogenous cardiomyocyte regeneration. New drug delivery approaches, high-throughput screening, as well as novel therapeutic compounds combined with gene editing will facilitate the development of potential cell-free therapeutics. In parallel, progress has been made in the field of cell-based therapies. Transplantation of human pluripotent stem cell (hPSC)-derived cardiomyocytes (hPSC-CMs) can partially rescue the myocardial defects caused by cardiomyocyte loss in large animals. In this review, we summarize current cell-based and cell-free regenerative therapies, discuss the importance of cardiomyocyte maturation in cardiac regenerative medicine, and envision new ways of regeneration for the injured heart.

## Introduction

Cardiovascular disease (CVD) remains a leading cause of morbidity and mortality globally. As cardiac regeneration is limited in adults, damaged cardiac regions form compensatory scars with very few functional cardiomyocytes, ultimately resulting in cardiac dysfunction and chronic heart failure. Current clinical therapies have been shown to enhance cardiac function, but none of them is designed to directly address the restoration of cardiomyocyte loss (1). Heart transplantation represents a standard treatment for patients with end-stage heart failure, however, the availability of organ donors is far from adequate to meet demand (2). It is therefore paramount to develop cardiac regenerative medicines.

Over the past two decades, fundamental advances have been made to uncover the cellular and molecular mechanisms of heart development (3, 4). The discovery of multiple signaling pathways and metabolic regulation of cardiac growth and homeostasis has shed light on potential endogenous mechanisms of cardiomyocyte regeneration. Novel drug delivery systems such as the adeno-associated virus 9 (AAV9) system or heart-targeted nanoparticles and the development of novel small molecules might allow for myocardial regeneration approaches in clinical settings (5–7). Moreover, human pluripotent stem cells (hPSCs)-derived cardiomyocytes (hPSC-CMs) have been extensively used for disease modeling and drug screening in CVD (8, 9). With the advancement of hPSC-CM research and cardiac organoid engineering, it has become possible to graft stem cell-derived-CMs into the injured heart, providing directions for optimizing these approaches. In this review, we list some candidates for cell-free regenerative therapy, discuss the transplantation of adult stem cells and hPSC-CMs in cell-based therapy, and envision new regenerative approaches to repair damaged hearts.

## Mechanisms underlying cardiac regeneration

**FIGURE 1:**
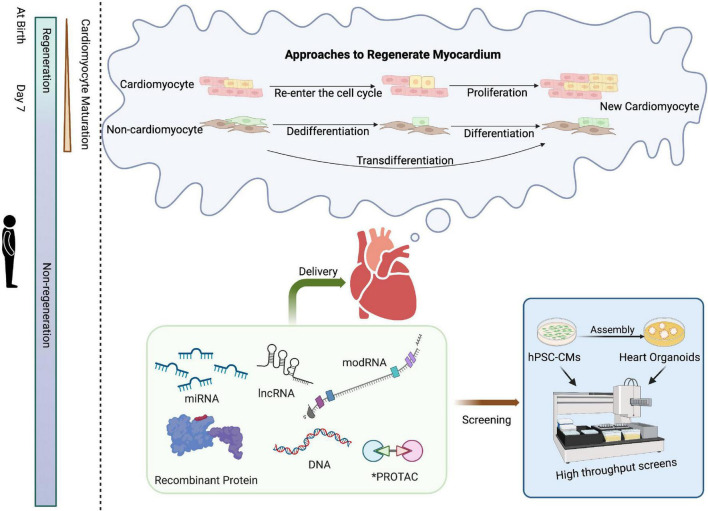
Schematic of approaches for cardiac regenerative medicine using cell-free therapies. Mammals have the intrinsic capability to structurally and functionally regenerate their hearts shortly after birth, a capacity that is subsequently lost. Approaches to cardiac regeneration involve the re-entry of cardiomyocytes into the cell cycle and/or transdifferentiation of other resident cell types into cardiomyocytes. Recombinant proteins, RNA-based drugs, PROTAC, or small molecules could serve as viable strategies for cardiac repair. High-throughput screening of drug candidates can be performed in hPSC-CMs or, at lower throughput, cardiac organoids prior to clinical application. Created with BioRender.com.

Although the adult heart has been shown to lack regenerative capacity in mammals (10, 11), the heart can effectively regenerate within the first week after birth. Studies of apical resection (12, 13) and left anterior descending (LAD) coronary artery ligation (14–16) in neonatal rodents have shown that murine, as well as rat cardiomyocytes, have an intrinsic regenerative capacity within the first 7 days after birth. Similarly, the neonatal porcine heart is capable of regeneration after acute myocardial infarction (MI) during the first 2 days after birth (17). Furthermore, we recently reported the complete functional recovery after a massive MI in a human newborn (18). Compared to the neonatal mammalian heart, adult mammalian cardiomyocytes are highly differentiated and often contain more than one nucleus and well-aligned sarcomeres to maintain cardiac function (19); however, this in turn hinders myocardial regeneration in the adult heart once the heart is damaged (Figure 1). Therefore, inducing mature cardiomyocytes to re-enter the cell cycle from a quiescent state is one of the strategies to repair damaged hearts.

To date, extensive studies of neonatal heart regeneration and adult heart repair following injury in mammals have identified fundamental mechanisms underlying cardiac regeneration, providing directions for repair after myocardial injury. The transcription factor GATA4 (20), for example, is known to play an essential role in cardiomyocyte replication in neonatal mice. Myocardial Erbb2 (21) and BMP (22, 23) signaling were found to control cardiomyocyte proliferation. Inhibition of adrenergic receptor (AR) and thyroid hormone (TH) pathways promoted cardiomyocyte regeneration in mice after postnatal day 7 (24). Activation of Neuregulin1/ErbB4 signaling (25) or overexpression of a single transcription factor, namely Tbx20 (22), promoted the repair of damaged adult cardiomyocytes after myocardial infarction in mice and enhanced cardiomyocyte cell-cycle entry. Deletion of Salvador, a component in the Hippo pathway, improved heart function after myocardial infarction (26). Moreover, deletion of the hypoxia response element Meis1 increased the number of cardiomyocytes, especially mononucleated cardiomyocytes in adult mice (27).

In addition to directly inducing cardiomyocyte proliferation, several studies have demonstrated that other cardiac cell types, such as fibroblasts, can transdifferentiate into functional cardiomyocytes, which may be a potential and viable approach to heart regeneration *in vivo*. A classic combination of transcription factors Gata4, Mef2c, and Tbx5 (GMT) enabled direct reprogramming of postnatal cardiac or dermal fibroblasts into spontaneously contracting cardiomyocyte-like cells with cardiac-specific markers and contracted spontaneously (28). One study showed that blocking TGF-β and WNT signaling increased the efficiency of reprogramming in GMT-overexpressing cardiac fibroblasts. *In vivo*, mice treated with GMT, TGF-β inhibitor SB431542, and WNT inhibitor XAV939 for 2 weeks after myocardial infarction significantly improved reprogramming and cardiac function compared to mice treated with GMT only (29). In addition, the transcription factor Tead1 (Td) could efficiently replace Tbx5 in the GMT cocktail, enhancing reprogramming efficacy (30). Such reprogramming can also be achieved by chemical induction alone. A combination of nine compounds induced the transdifferentiation of fibroblasts into contracting cardiomyocyte-like cells (31). Importantly, fibroblasts can be directly reprogrammed to spontaneously contracting patches of differentiated cardiomyocytes without a pluripotent intermediate through transgenic expression of Oct4, Sox2, Klf4, and c-Myc (32). Recent studies have shown that in addition to fibroblasts, endocardial cells have the potential to generate cardiomyocytes (33). For example, the deletion of the stem cell leukemia (SCL) gene induces the expression of cardiac-specific proteins in endothelial cells (34).

Numerous studies have uncovered mechanisms that promote cardiac regeneration, and artificially increasing or decreasing these critical molecules *in vivo* may alleviate or even rescue the pathogenesis heart disease process. Thus, the discovery of druggable regenerative targets is vital to cell-free therapies.

## Cell-free therapies

For cardiac repair, recombinant DNA, RNA-based, or recombinant protein therapeutics have been used in regenerative medicine. Here, we discuss some potential drug/molecule candidates for cell-free therapies based on preclinical reports of cardiac regeneration (Table 1).

**TABLE 1:** Potential targets and candidates for cardiac regenerative cell-free therapies.

Candidates	Regulation	Application		Outcome	References
Tbx20	Up	Transgenic mice	Mice	Promotes cardiomyocyte proliferation	(22)
mir302-367	Up	Systemic delivery of miRNA	LAD mice	Induces cardiomyocyte proliferation and promotes cardiac regeneration post MI	(128)
miR-31a-5p	Up	miR-31a-5p antagomir	Neonatal rat	Promotes postnatal cardiomyocyte proliferation	(129)
NRG1	Up	Injection of NRG1 protein	LAD mice	Induces cardiomyocyte proliferation and promotes myocardial regeneration following MI	(25)
Jagged1	Up	–	–	Notch activation promotes immature cardiac myocyte proliferation and expansion at early time points in neonatal rats	(130)
GATA4	Up	–	–	GATA4 directly interacts with Cyclin D2 and Cdk4 promoters in cardiac myocytes from mice	(131)
CDK1, CDK4, Cyclin B1 and Cyclin D1	Up	Delivery of recombinant CDK1, CDK4, cyclin B1 and cyclin D1	LAD mice	Enhances cardiac function in mice after acute or sub-acute MI	(36)
Cyclin A2	Up	Adenoviral vector delivery	LAD rat	Induces cardiomyocyte mitotic activity and improves ventricular function after ischemic injury	(35)
IGF-1, HGF	Up	Administration of recombinant IGF-1/HGF	Intracoronary balloon occlusion in pigs	Improves cardiac function following MI	(44)
FGF16	Up	AAV9 delivery	Neonatal Gata4fl/fl mice with Cryoinjury	Rescues cryoinjury-induced cardiac hypertrophy and improved heart function after injury	(20)
Pkm2	Up	Delivery of Pkm2 modRNA	LAD mice	Increases cardiomyocyte cell division and improves cardiac function following MI	(41)
Agrin	Up	Recombinant Agrin	LAD mice	Stimulates cardiomyocyte proliferation in primary cardiac culture and is involved in cardiac regeneration in neonatal mice	(132)
PPARδ	Up	PPARδ agonist	LAD mice	Improves heart function in mice after myocardial infarction	(133)
hsa-miR-590, hsa-miR-199a	Up	AAV9-miRNA	Neonatal rat	Promotes cardiomyocyte proliferation in adult mice and improves cardiac function following MI	(134)
Hypoxia	Up	Hypoxia condition	LAD mice	Induces cell cycle re-entry of adult cardiomyocytes and improves functional recovery following MI in adult mice	(42)
ERBB2	Up	Transgenic mice	Erbb2-cKO mice	Transient induction of ERBB2 in adult mice is sufficient to reactivate CMs to proliferative and induce their regenerative potentials after ischaemic injury	(21)
FSTL1	Up	Patch with FSTL1 to the epicardium	LAD mice and pig	Stimulates cell cycle entry of CMs and improves cardiac function and survival in mouse and swine models of myocardial infarction	(46)
Yap1	Activated	–	–	Stimulates proliferation of postnatal cardiomyocytes in mice and in cultured rat cardiomyocytes	(135)
Gata4, Mef2c and Tbx5 (GMT)	Up	Injection of GMT-encoding retrovirus	LAD mice	Enhances cardiac reprogramming and cardiac function	(29, 43)
miR-99/100, Let-7a/c	Down	AAVs encoding for anti-miR-99/100 and anti-Let-7a/c	LAD mice	Adult cardiomyocyte dedifferentiation, enhances cardiomyocyte proliferation, and facilitates heart regeneration	(39)
LncDACH1	Down	Adv-LncDACH1, or Adv-shLncDACH1	LAD mice	Stimulates cardiac regenerative potential and enhanced cardiac function in the injured heart	(40)
LrP6	Down	AAV9-miRNAi-Lrp6 delivery	LAD mice	Reduces scar size in the infarcted hearts of mice and stimulates cardiomyocyte proliferation in the infarct border zone	(37)
Meis1	Down	Deletion of *meis1 in* mice	Adult mice	Induces cell cycle re-entry in mice	(27)
FGF1, p38 MAP kinase	Down	FGF1/p38 inhibitor	LAD rat	Induces cardiomyocyte proliferation and rescue cardiac function following MI	(136)
Dag1	Down	–	TAC mice	The dystrophin– glycoprotein complex component dystroglycan 1 (Dag1) directly binds to the Hippo pathway effector Yap to inhibit cardiomyocyte proliferation in mice	(137)
α-catenins	Down	Gene depletion	αE- and αT-Catenin double KO mice	Leads to nuclear accumulation of Yap and induction of cardiomyocyte proliferation in mice	(138)
GSK-3β	Down	Gene depletion	GSK-3β conditional KO mice	Protects against post-MI remodeling and promotes cardiomyocyte proliferation in adult mice	(139)
GHRH-A	Down	Injection of a hormone-releasing hormone agonist (GHRH-A)	LAD pig	Reduces infarct size and improve cardiac function in pigs with subacute ischemic cardiomyopathy	(45)
Adrenergic receptor (AR), thyroid hormone (TH)	Down	AR and TH inhibitors	Neonatal mice	Extends postnatal cardiac regenerative capacity in part by promoting cardiomyocyte cell division	(24)

In murine MI models, for example, injection of Neuregulin1 induced a sustained improvement in myocardial function and attenuated compensatory hypertrophy following MI (25). Adenoviral-based delivery of cyclin A2 increased myofilament density at the border zone of the MI and improved cardiac function (35). Moreover, cardiac-specific overexpression of FGF16 via AAV subtype 9 (AAV9) led to an upregulation of genes associated with cell proliferation in *Gata4*-ablated mouse hearts (20). Combined intramyocardial injection of CDK1/CCNB/CDK4/CCND significantly improved ejection fraction (EF), stroke volume, cardiac output, and markedly reduced the scar size (36). Down-regulation of Lrp6, a Wnt co-receptor, promoted adult post-MI cardiac repair by increasing cardiomyocyte proliferation (37). Delivery of IGF2BP3 through AAV9-Igf2bp3 into neonatal mouse hearts 3 days prior to LAD ligation significantly improved heart function as determined at 3-weeks post-injury (38). Some RNAs are potential targets for cardiac regeneration. For example, silencing miR-99/100 and Let-7 can induce cardiomyocyte dedifferentiation and improve heart function in adult LAD-treated mice (39). Knockdown of LncDACH1 using LncDACH1 shRNA (Adv-shLncDACH1) reactivated cardiomyocyte proliferation in adult mice and enhanced cardiac function in the injured heart (40). Delivery of Pkm2 modified RNA (modRNA) in mice hearts can increase cardiomyocyte cell proliferation and improve cardiac function after myocardial infarction (41). Moreover, one study showed chronic hypoxia-induced cardiac regeneration in adult mice. Long-term low oxygen treatment induced cardiomyocyte proliferation and angiogenesis *in vivo*, thereby reducing myocardial fibrosis and improving left ventricular systolic function in mice with myocardial infarction (42). In addition, induction of non-cardiomyocyte transdifferentiation into cardiomyocytes *in vivo* can also be achieved. Direct intramyocardial injection of GMT transdifferentiated non-cardiomyocytes into new cardiomyocyte-like cells, decreased infarct size, and attenuated cardiac dysfunction after myocardial infarction in mice hearts (43).

In swine MI models, cardiomyocyte hypertrophy and fibrosis following chronic MI were reduced when IGF-1/HGF was intramyocardially delivered into the injured area (44). Subcutaneous injection of a daily dose of growth hormone-releasing hormone agonist (GHRH-A) into pigs with a LAD ligation showed left ventricular structural and functional improvements, whereas cardiomyocyte proliferation was not significantly altered (45). In addition, the cardiomyogenic factor Follistatin Like 1 (FSTL1), produced by the epicardium, can stimulate recovery of contractile function within 2 weeks and limit fibrosis 4 weeks after MI injury, suggesting that FSTL1 has therapeutic efficacy in a large animal MI I/R swine model (46).

Although there many targets for cardiac regeneration have been identified and validated in animal models, the drugs currently available for clinical application are limited. The development of human cardiomyocytes from pluripotent stem cells will undoubtedly help test delivery systems and screen novel drugs for cardiac regeneration in a human “background” since hPSC-CMs from patients can also be used for preclinical tests for drug toxicity, thus enabling more precise and personalized treatments (47, 48). For example, one study designed an engineered bivalent neuregulin-1β that attenuates doxorubicin-induced double-strand DNA breaks in hPSC-CMs, with the vision to utilize such treatment to protect the heart from doxorubicin cardiotoxicity (49). hPSC-CMs from Arg663His-mutated patients can be treated with the L-type Ca2+ channel blocker verapamil to avoid the development of the hypertrophic cardiomyopathy phenotype *in vitro*. Therefore, verapamil might be a potential drug for patients with Arg663His-mutated hypertrophic cardiomyopathy (50). Compared with 2D hPSC-CMs, human cardiac organoids generated from human pluripotent stem cells through cell self-assembly (51) and 3D printing (52) are more similar in the structure and function of the human heart. Combined with gene editing, these 3D tissues can now be used to model various cardiovascular diseases such as myocardial infarction (51) and thus can ultimately be used as models for screening a collection of drug candidates (Figure 1).

## Cell-based therapies for cardiac regeneration

Heart transplantation is currently the only restorative therapy for end-stage heart failure patients. Although the development of new drugs and surgical as well as improved storage techniques have led to an increase in successful heart transplantations, heart transplantation is still a high-risk medical procedure, and there remains an insufficient amount of donor hearts. In addition, immunosuppression is required after heart transplantation, which is a risk factor for complications. In recent years, cell-based therapies have been proposed as a promising approach for treating advanced heart failure and repairing damaged myocardial tissue.

## Adult stem cells transplantations

Early evidence suggested that adult stem cells such as bone marrow cells (BMCs), bone marrow-purified haematopoietic stem cells (HSCs), and bone marrow-purified mesenchymal stem cells (MSCs) can differentiate into cardiomyocytes. A 2001 study showed that 9 days after transplantation of c-kit+ BMCs in a LAD mouse model, newly formed myocytes occupied 68% of the infarcted region in the ventricle leading to an overall improvement in cardiac function (53). Then, one report claimed that the grafts of c-Kit+, stem cell antigen-1 positive (Sca-1+) BMCs migrated to ischemic areas where they differentiated into cardiomyocytes and endothelial cells (54). C-kit+ cells (55) and Sca-1+ cells (56, 57)were hence considered as adult cardiac stem/progenitor cells (CPCs). However, multiple follow-up studies showed negative results (58, 59). One study found that transplantation of HSCs into adult mouse hearts did not result in any detectable transdifferentiation into cardiomyocytes, nor was there a significant increase in cardiomyocytes in the HSCs-treated hearts (58). Likewise, multiple laboratories have demonstrated that the transplantation of c-kit+ cells into infarcted adult mouse hearts did not result in the differentiation of cardiomyocytes (60, 61). Additional studies further showed that Sca-1+ cells do not generate new cardiomyocytes (62–64), but are rather precursors of endothelial cells (62). Moreover, lineage-tracing techniques have confirmed that both c-kit+ and Sca1+ adult stem cells in transplanted mice cannot differentiate into cardiomyocytes *in vivo* (62–67). Thus, the concept of adult cardiac stem cells, as well as the idea that adult stem/progenitor cells can promote cardiac remuscularization, have been rejected.

Nonetheless, numerous clinical trials of bone marrow-derived adult stem cell transplantation have been conducted [reviewed in (68–70)]. As expected from foundational research, the overall clinical benefit was not significant. To date, there is growing evidence that the minute benefits of adult stem cell therapy could be attributed to the effects of secreted factors acting on neighboring cells through a paracrine mechanism (69, 70). Several key secreted growth factors have been identified, such as VEGF, HGF, IGF-1, and TGF-β, mediators that stimulate angiogenesis, inhibit apoptosis or modulate inflammatory pathways (71, 72). In addition, exosomes might be one of the reasons for the improvement of cardiac function after such adult stem cell transplantation. Treating the infarcted area with exosomes secreted by cardiac mesenchymal stem cells can enhance cardiac angiogenesis, promote cardiomyocyte proliferation, and maintain cardiac function in mouse hearts (73). In addition, one study found that both live and dead adult stem cells induced macrophage accumulation in the infarcted area of hearts, improving the heart function after I/R injury, which also occurred after the direct induction of innate immune response. Thus, the recovery of the infarcted area of the heart following adult stem cell therapy may attribute to an acute inflammatory wound-healing response through the accumulation of regional macrophages (74).

## Pluripotent stem cell-based therapies for cardiac regeneration

Human embryonic stem cells (ESCs) have the ability to differentiate into multiple cell types and thus have great therapeutic potential in regenerative medicine. However, because human ESCs are extracted from blastocysts, both scientific research and clinical applications of human ESCs face ethical issues (75). In 2006, Takahashi and Yamanaka successfully induced pluripotent stem cells (iPSCs) from fibroblasts by the introduction of four factors, Oct3/4, Sox2, c-Myc and Klf4. The self-renewal and differentiation capacity of pluripotent stem cells is largely comparable to that of embryonic stem cells but avoids ethical issues (76). In recent years, many laboratories have reported the development of cardiomyocytes from ESCs (77, 78) and iPSCs (79–84). ESC-derived cardiomyocytes (ESC-CMs) and iPSC-derived cardiomyocytes (iPSC-CMs), here collectively referred to as hPSC-CMs, express molecular markers and exhibit subcellular structures and electrophysiology resembling primary, albeit immature cardiomyocytes.

Several groups have transplanted hPSC-CM in experimental cardiovascular disease models *in vivo* (85–93), providing experimental feasibility studies for future clinical applications (Table 2). Studies have confirmed that hPSC-CMs can engraft, survive, and electrically couple with host myocardial tissue *in vivo* and improve contractile function after infarction. For example, in both acute myocardial infarction and chronic post-infarction heart disease in rats, transplanted hPSC-CMs can survive and form viable tissue containing striated cardiomyocytes. These hPSC-CM injections attenuated ventricular dilatation and preserved systolic function after acute myocardial infarction but are insufficient to alter adverse remodeling of chronic myocardial infarction rats (90, 91). In addition, transplanted hPSC-CMs could remuscularize cryoinjured guinea-pig hearts, thereby preserving cardiac function (92). Intramyocardial delivery of one billion hPSC-CMs into Macaques suffering an ischemia/reperfusion injury also resulted in the remuscularization of substantial areas of the infarcted monkey heart (93). The hPSC-CM engraftment is indeed promising as a cell-based therapy. However, there are key issues that remain to be solved.

**TABLE 2:** Preclinical and clinical studies of hPSC-CMs transplantations for treatment of cardiac disease.

Species	Disease model	Cell types	Delivery method	Heart function	Side effect	References
Mice	LAD	hiPSC-CMs	Intramyocardial injection	Enhance	No major side effects reported	(85, 87)
Rat	I/R	hESC-CMs	Intramyocardial injection			(90)
	LAD	hiPSC-CMs	Intramyocardial injection			(86)
	LAD	hiPSC-CMs and rat microvessels	Intramyocardial injection			(111)
Guinea-pig	Cryoinjury	Partly matured hESC-CMs	Intramyocardial injection		Arrhythmia but reduced	(99)
Pig	Ameroid ring placement	hiPSC-CMs	Cell sheet		Arrhythmia	(88)
	LAD	hESC-CMs	Direct image-guided transendocardial injection			(95)
	Cryoinjury	hiPSC-cardiac spheroids	Intramyocardial injection			(110)
Monkey	I/R	hESC-CMs	Intramyocardial injection			(93)
	LAD	hESC-CMs				(97)
	LAD	mPSC-CMs				(94)
Human	Patients	hiPSC-CMs	Injection	Not yet reported	Not yet reported	(120)
		hiPSC-CMs	Patches	Clinical symptoms improved	No adverse events	(114)

Arrhythmias are considered the most critical side effect of engraftment, as they can be lethal, especially in pigs and primates. In ischemia/reperfusion-injured macaques, ventricular arrhythmias were observed despite remuscularization (93). Similar results were found in myocardial-infarcted cynomolgus monkeys. Ventricular tachycardias happened following the transplantation of monkey iPSC-derived cardiomyocytes (mPSC-CMs) (94). Studies in infarcted hearts of rats and pigs also showed the development of arrhythmias and tachyarrhythmias following injection of immature hPSC-CMs (95, 96). In infarcted hPSC-CM recipient pigs, frequent and fatal ventricular tachyarrhythmias were observed during the first few days of post-transplantation, and normal sinus rhythm was observed 28 days after transplantation (95). Such graft-related ventricular arrhythmias most likely originate from an ectopic pacemaker formed by the transplanted hPSC-CMs (97). To eliminate such arrhythmic events, several strategies have been considered. Pharmacologic treatment is one of the solutions to engraftment arrhythmia. One study showed that a combination of amiodarone and ivabradine could effectively suppress arrhythmia in infarcted hPSC-CM recipient pigs (98). In addition, the engraftment of more mature cardiomyocytes was beneficial in reducing arrhythmia events (99). This study showed that hPSC-CMs cultured on polydimethylsiloxane (PDMS) substrates exhibited increased expression of cardiac maturation markers and improved structural and functional properties of more mature cardiomyocytes *in vitro.* They then found that transplantation of this PDMS-treated hPSC-CMs in an infarcted guinea pig enhanced post-transplant structure and alignment, host-graft electromechanical integration, and importantly, reduced proarrhythmic behavior (99). To engraft matured hPSC-CMs, several studies have attempted to induce cardiomyocyte maturation *in vitro*. For example, using 3–6 months long-term cultures, hPSC-CMs exhibited an adult-like phenotype, including increased cell size or greater myofibril density and alignment (100, 101). In addition, electric pacing and mechanical stimulation were shown to promote hPSC-CMs maturation *in vitro* (102, 103). hPSCs-CMs treated with a maturation medium including a peroxisome proliferator-activated nuclear receptors alpha (PPARa) agonist, palmitate, dexamethasone, and Tri-iodo-l-thyronine (T3) (104) in the presence of low glucose resulted in hPSC-CMs with increased the expression of genes associated with fatty acid oxidation (FAO), mitochondrial respiration, and muscle function (105). In addition, insulin-like growth factor-1 (IGF-1) or low glucose in culture media was shown to promote cardiomyocyte maturation (106, 107). In contrast to monolayer cardiomyocyte cultures, hPSCs-CMs grown in 3D *in vitro* appear to be more mature and thereby better mimic bona fide cardiomyocytes (108). In particular, self-organizing cardiac organoids, as compared to 2D-grown hPSC-CMs, exhibit increased expression of cardiac ion channels (KCNH2), structural proteins (TNNI1, TTN, and MYH6), cardiac transcriptional factors (TBX5 and MEF2C), or sarcoplasmic reticulum proteins (RYR2 and ATP2A2), indicating improved maturity (108).

During the transplantation of exogenous hPSC-CMs, the nutrient-deprived and hypoxic environment in the infarcted area is a major challenge (109). Although studies demonstrated that hPSC-CMs could be engrafted in monkey hearts and survive up to 3 months (93, 94, 97), another report found that the engrafted hPSC-CMs were massively reduced in numbers after 8 weeks post-transplantation in pig hearts (110). Therefore, the addition of support cells may be beneficial for hPSC-CMs integration and survival. Indeed, co-transplantation of microvessels and hPSC-CMs into the ischemic area of the LAD-treated rats promoted the survival of hPSC-CMs *in vivo* and improved cardiac function compared with the transplantation of hPSC-CMs alone (111). Although the mechanisms involved in the functional integration and survival of hPSC-CMs in host tissues are not fully understood, studies have found vascularization occurs after hPSC-CMs transplantation and may be related to cytokines such as VEGF secreted by the grafted cells (109, 112). Therefore, the addition of VEGF (113) or other pro-angiogenic factors before transplantation may also contribute to the improvement of hPSC-CMs survival and subsequent enhanced cardiac function.

Besides cardiomyocyte maturation and vascularization, the mode of delivery might be critical. Intracardiac injection is the current delivery method, but grafts may be eluted with the circulatory system. To enhance hPSC-CMs survival, a multicomponent pro-survival cocktail was developed, and its co-injection with hPSC-CMs improved graft residency *in vivo* (90, 91). Bioengineering methods such as cell patches (114, 115) and cell sheets (116, 117) have also been devised to improve cell engraftment rates, however, integrating cells in biomaterials with host myocardium is a big challenge. For example, transplantation of hPSC-CMs sheets improved cardiac systolic function not attributable to graft integration into the host myocardium but most likely due to neovascularization (118). Recently microneedle patches were developed to be inserted into the myocardium, improving the connection between the graft and the host myocardium (119).

**FIGURE 2:**
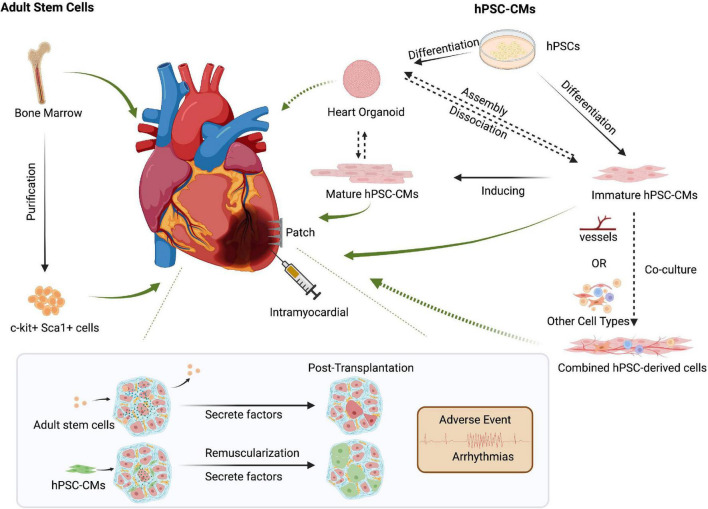
Cell-based approaches to cardiac regenerative medicine. Delivery methods such as intracardiac injection and cell patches can be used for cell-based therapies. Though controversial, transplanting bone marrow-derived adult stem cells could promote cardiac function via secreted factors. Human pluripotent stem cell-derived cardiomyocytes (hPSC-CMs) can repair damaged hearts through tissue replacement of lost cardiomyocytes and help promote cardiac function by secreting growth factors such as VEGF. However, preclinical models and clinical trials must carefully address post-transplant arrhythmias and other side effects. The increased maturity of hPSC-CMs might reduce unwanted and potentially lethal arrhythmic events. Co-delivery of multiple cell types, including endothelial cells or other cardiac cell types, might improve hPSC-CMs retention and thereby promote the repair of injured hearts. Created with BioRender.com.

So far, there have been two clinical trials engrafting hPSC-CMs for heart disease. Two patients in China underwent an experimental treatment for heart disease based on hPSC-CMs, though the clinical outcomes have not yet been published (120). In Japan, one male patient who suffered from severe heart failure due to ischemic cardiomyopathy was treated with clinical-grade hPSC-CMs patches. The clinical symptoms apparently improved 6 months after surgery, without any major adverse events or changes in the cardiac wall motion at the site of the transplant. However, more details need to be disclosed (114). Regardless, these first human clinical trials hold promises for the use of hPSC-CMs to repair cardiac damage (Figure 2).

## Future directions and conclusion

The field of cardiac regeneration has made remarkable progress in recent years. Both cell-free and cell-based methods are vigorously researched and developed to promote and improve cardiac regeneration for clinical applications. Along the way, numerous molecular mechanisms and key factors involving cardiomyocyte’s re-entry into the cell cycle or trans-differentiation of non-cardiomyocytes into cardiomyocytes were discovered and are now being translated to drug development. Although some molecules, such as recombinant proteins, small molecule inhibitors, or RNA-based therapies, are being developed, more effective drugs need to be discovered. Moreover, Proteolysis Targeting chimera (PROTAC) technologies might provide viable modes of drug delivery for targeted and time-resolved degradation of candidate drug targets (121, 122).

For cardiac repair using cell-based systems, hPSC-CMs have the potential to form functional tissue containing striated cardiomyocytes *in vivo.* To achieve clinical use, hPSC-CMs will be required to be mass-produced with strict quality standards. Therefore, allogeneic, off-the-shelf hPSCs-CMs must be developed. In addition to pharmacological immunosuppression, including new-generation drugs with fewer side effects, gene-edited hypoimmune hPSC-CM have been generated to overcome the rejection from the host (123). Another obstacle is the maturity of transplanted hPSC-CMs, in particular, addressing and reducing arrhythmic events triggered by the transplanted cardiomyocytes that have to be functionally integrated into the electrically coupled cardiac tissue. Compared to monolayer cultures, 3D hPSCs-CMs appeared to express more maturation markers and functionally mimic more mature cardiomyocytes, including the formation of tight junctions between cardiomyocytes. Thus, transplantation of hPSC-CM aggregates rather than loose single cardiomyocytes may contribute to graft survival, improve functionality and reduce arrhythmias. However, several studies suggest that the optimal timing of transplantation depends on the developmental stage of hPSC-CMs (124, 125). Moreover, the mode of delivery of such cell-based therapies will be critical. Balancing hPSC-CMs maturity, effective delivery, and transplantation timing must be the focus of future research.

Besides cardiomyocytes, the heart contains multiple other cell types, such as endothelial cells, fibroblasts, smooth muscle cells, or different types of immune cells, that might affect graft survival and improve the function of damaged hearts (126). As more hPSC-derived cell types can be faithfully generated, co-transplantation of multiple cell types might therefore greatly improve cell-based therapies for cardiac diseases. For instance, our group developed stem cell-derived self-organizing 3D blood vessel organoids (BVOs) that form bona fide and functionally perfused vascular trees containing arterioles, capillaries, and venules when transplanted into immunodeficient mice (127). Such BVOs and other approaches to generate human endothelial cells and blood vessels, such as 3D printing, could be utilized to enhance and maintain the engraftment of stem cell-derived cardiomyocytes.

## Author contributions

JW wrote and revised the draft. JP designed and supervised and revised the study. MA designed the tables. BH revised the draft. All authors read and approved the submitted version.
